# RNA Interference in Insect Vectors for Plant Viruses

**DOI:** 10.3390/v8120329

**Published:** 2016-12-12

**Authors:** Surapathrudu Kanakala, Murad Ghanim

**Affiliations:** Department of Entomology, The Volcani Center, Rishon LeZion 7505101, Israel; kanakalavit@gmail.com

**Keywords:** RNAi, dsRNA, plant viruses, insect vectors, insect pest control, virus induce gene silencing (VIGS)

## Abstract

Insects and other arthropods are the most important vectors of plant pathogens. The majority of plant pathogens are disseminated by arthropod vectors such as aphids, beetles, leafhoppers, planthoppers, thrips and whiteflies. Transmission of plant pathogens and the challenges in managing insect vectors due to insecticide resistance are factors that contribute to major food losses in agriculture. RNA interference (RNAi) was recently suggested as a promising strategy for controlling insect pests, including those that serve as important vectors for plant pathogens. The last decade has witnessed a dramatic increase in the functional analysis of insect genes, especially those whose silencing results in mortality or interference with pathogen transmission. The identification of such candidates poses a major challenge for increasing the role of RNAi in pest control. Another challenge is to understand the RNAi machinery in insect cells and whether components that were identified in other organisms are also present in insect. This review will focus on summarizing success cases in which RNAi was used for silencing genes in insect vector for plant pathogens, and will be particularly helpful for vector biologists.

## 1. Introduction

Insect vectors for plant pathogens are worldwide pests and pose a continuous threat to plants of economic importance. They vector bacteria and fungi and are responsible for the transmission of more than 70% of all known plant viruses [1]. The damage caused by plant viruses is estimated in the billions of dollars each year globally. The majority of insect vectors are controlled using chemical insecticides that threaten the environment, human health and beneficial organisms. More importantly, the development of insecticide resistance among many insect pests especially vectors for pathogens is a common problem. Therefore, the development of effective and non-chemical control methods against insect vectors is a major challenge. The development and use of genetically modified resistant plants have emerged as an important strategy that has proven to be important for the management of insect pests. Notably, two approaches have been developed: the first involves the use of transgenic plants expressing toxins from the bacterium *Bacillus thurengiensis* (Bt), and the second approach using plant-derived genes such as those that encode for inhibitors or lectins. However, many factors still limit these approaches to be fully successful such as: (a) Bt crops are not universal to all insect pests and are limited in their effectiveness against all insect; (b) sustainability; and (c) durability. Combining this with the increased ability of insect pests to develop resistance to all major insecticides including resistance to transgenic plants have facilitated the development of new methods for plant protection including RNAi-based protection.

RNAi is still considered as one of the most significant discoveries in biological research, and used for functional analysis of genes in eukaryotic organisms, including plants, mammals and insects. RNAi was first discovered in the worm *Caenorhabditis elegans* [2], in plants [3] and insects, first in the fruitfly *Drosophila melanogaster* [4]. The mechanisms and components of the RNAi machinery have been identified in many insect species including the functional analysis of genes in model and non-model insects [5,6]. During the last decade, many research projects have employed RNAi and made it possible to unveil the function of new genes and developed a new research discipline in which the development of pesticide-free control methods against insect pests became realistic. More importantly, recent results have suggested that insect vectors for plant pathogens bear the RNAi machinery as it was discovered in model organisms, leading to the hypothesis that vector-borne pathogens could be managed by silencing essential genes in the vectors either by interfering with the transmission or by killing the insect vector itself. Such approaches have not been employed for controlling vectors of plant pathogens, however more successful cases have been reported for suppressing essential genes that influence the insect fitness and fecundity [6]. The purpose of this review is to highlight successful cases in which RNAi was used for controlling or limiting populations of vectors of plant pathogens.

## 2. Mechanism of RNA Interference in Insects

RNA interference is a mechanism of post-transcriptional gene regulation in eukaryotes [7]. This process involves the synthesis of double stranded RNA (dsRNA) which is cleaved by the ribonuclease III type Dicer [8,9] into 20–25 bp small RNAs in an ATP dependent manner [3,10]. These small RNAs comprise three distinct groups, short-interfering RNA (siRNA, 19–24 nucleotides), microRNA (miRNA, 19–24 nucleotides), and piwi-interacting RNA (piRNA, 24–30 nucleotides). These fragments are assembled by Argonaute proteins to form a multi-protein RNA-induced silencing complex (RISC) that recognizes and targets the destruction of complementary gene or messenger RNA [11,12].

**Figure 1:**
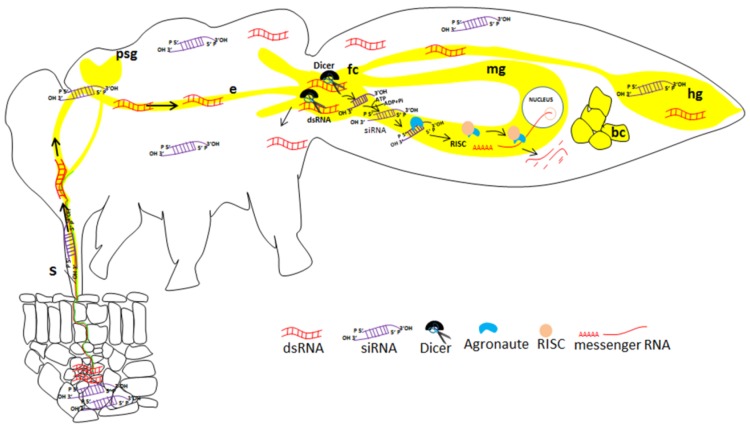
Illustration of gene silencing in a sap-sucking insect (*Bemisia tabaci*) by plant-mediated acquisition of siRNA. The siRNA molecules are acquired from the plant vascular tissue, move along the food canal and cross the midgut to the hemolymph, where they can reach many tissues in which silencing may be induced. The midgut is the first tissue in which efficient silencing may occur. S: stylet; psg: primary salivary glands; e: esophagus; fc: filter chamber; mg: midgut; hg: hindgut; bc: bacteriocyte.

The effects of RNAi responses can be well categorized into, intracellular and extracellular RNAi. The intracellular RNAi involves the introduction of dsRNA into cells using delivery methods such as injection, transfection or electroporation directly into a cell. Extracellular RNAi involves delivery by injection, soaking or feeding [13,14,15,16,17]. The RNAi mechanism has been further categorized into two types: non-cell-autonomous and cell-autonomous. In cell-autonomous RNAi, the silencing process is limited to the cell in which the dsRNA is introduced [18]. Here, the dsRNA of the target gene is processed into 21–24 nt duplexes by RNase (Dicer) and then, these siRNAs are incorporated into RISC, which mediates mRNA degradation (Figure 1).

Non-cell-autonomous RNAi takes place mostly in multicellular organisms, for example, *C. elegans* [2]. This type involves an RNAi-mediated knockdown of the target gene expression at a different site from the production or application site of the dsRNA. There are two types of non-cell-autonomous RNAi: environmental RNAi and systemic RNAi. Environmental RNAi has been observed in a wide range of species, in which dsRNA is taken up by a cell from the environment. It was first discovered in *Drosophila* S2 cells [19,20,21]. Systemic RNAi Defective (SID-1) protein was shown to be involved in extending the silencing signal from the cell/tissue in which the dsRNA is applied to other cells/tissues [22]. SID-1 is essential and sufficient to mediate systemic spread of RNAi signal in both somatic and germ-line cells [23,24].

## 3. Delivery Methods of RNAi Molecules

The success of an RNAi experiment depends upon the selection of: (1) target gene; (2) length range of dsRNA; and (3) delivery method. For efficient RNAi, target gene silencing should significantly influence the insect’s fitness or cause mortality. The length of the target gene also influences the uptake and silencing efficiency. Efficient knockdown further depends on the concentration of dsRNA and delivery method. Several previous reports have proposed a size range between 50 and 520 bp as the limit in inducing effective silencing [25,26,27,28,29,30,31,32,33]. Several previous studies have developed dsRNA delivery methods for introducing dsRNA into the insect hemocoel using microinjection with varying concentrations [34,35,36,37,38]. Other methods included feeding with dsRNA through artificial diet/oral delivery [39,40,41]; feeding with bacteria that expresses dsRNA [42], soaking in dsRNA [43]; nano-particle-mediated RNAi [44] and fungi expressing the specific dsRNA [45]. These methods were adapted for studying RNAi mechanisms in insects for research purposes, but none could be suggested as strategy for pest control under field conditions.

Engineering crops with insecticidal antimetabolic protein or plant derived genes toxic to insects can be an alternative defense strategy to control insect herbivory, growth, development and in some cases cause the death of the insect. It has been suggested that hairpin RNA (hpRNA) expressed in planta, were processed into siRNA by the plant Dicer and those siRNA were detected in the phloem sap of the transgenic plants [46,47]. There are three main approaches to obtain transgenic plants resistant to insects. The first involves the use of Bt delta-endotoxins, the second approach uses plant-derived genes, such as enzyme inhibitors or lectins [48,49], and the third involves plants expressing dsRNA/siRNA that target insect genes. Three approaches can be followed to generate such plants for silencing: (a) Stable transgenic plants expressing dsRNA [14,15]. The transgenic plants expressing specific dsRNA against insect genes have provided promising results, however none of these reached the level of commercialization for controlling insects under field conditions. (b) Virus induced gene silencing (VIGS) to transiently silence target genes of insects feeding on host plant [32,50]. In this regard, it was suggested that the phloem of virus-infected plants contains high level of siRNA [51]. This indicates that siRNA could be acquired by insects especially sap-sucking insects, following VIGS. (c) Spray of dsRNA on plants that could be acquired by the insects to induce silencing [43]. Developing such methods for the control of vectors of plant pathogens is scarce; however, VIGS and dsRNA spray have the potential to serve this goal.

## 4. RNAi-Based Approaches for the Control of Insect Vectors

To date, RNAi has been documented and studied in about thirty insect species that belong to nine insect orders [6]. Insect vector for plant pathogens can be found in seven of the 32 orders in the Insecta [52]. However, among these, sap-sucking insects that include aphids, whiteflies, hoppers, thrips and beetles are the major vectors that transmit the highest diversity and most important plant pathogens. For silencing in insect vectors, two approaches could be undertaken: one approach is silencing target genes for inducing mortality and controlling the insect population and the second is silencing that could lead to interference with the transmission. The cases that were reported in the different insect species will be summarized hereafter (Table 1).

### 4.1. Aphids

Aphids are the largest group of insects that transmit plant viruses and it is estimated that 28% of all plant viruses that belong to eight families, 18 genera, and some taxonomically unassigned viruses are transmitted by aphids [1,52]. RNAi has been successfully applied in aphids and silencing of aphid genes has shown significant effects on the insects such as mortality and reduction in fecundity.

The pea aphid *Acyrthosiphon pisum* is an important pest and transmits both non-persistent and persistent plant viruses [53]. In *A. pisum*, RNAi-mediated gene silencing was first shown by silencing the *C002* gene [35]. Thereafter, other genes were targeted for silencing including *C002*, *Mp10* and *Mp42* [54], *Calreticulin* [36], *vATPase* [38], *Aquaporin*, *ApAQP1* [40], gap gene hunchback (*hb*) [29], *cathepsin*-*L* [55], Angiotensin-converting enzymes (*ACEs*) [56], structural sheath protein (*shp*) [57], Peroxiredoxin 1 gene (*ApPrx1*) [58], macrophage migration inhibitory factor (*MpMIF1*) [59] and *Cry4Aa* derived from *B. thuringiensis* subsp *israelensis* [60]. Among these, knockdown of *C002* and *Cathespsin*-L *vATPase*, *ACEs*, *MpMIF1* and *shp* genes showed significant mortality of *A. pisum*.

The peach potato aphid *Myzus persicae* is probably the most important aphid species as virus vector and transmits many important plant viruses including Potato virus Y (PVY), Potato leafroll virus (PLRV) and Cucumber mosaic virus in many regions of the world [53]. Thus far, about 17 genes showed significant effects on the insect after silencing. For example, *M. persicae* feeding on the transgenic plants producing the dsRNAs or siRNA of salivary proteins *MpC002*, *MpPIntO1*, and *MpPIntO2*; receptor of activated kinase C gene *MpRack1* [46,61,151]; serine protease (*MySP*) [62]; Acetylcholinesterase 2 gene *MpAChE2*; *V-ATPase E*; 40S ribosomal protein S5-like isoform-1 *Rps5*; SWI/SNF-related matrix-associated actin-dependent regulator of chromatin subfamily D member 1-like gene *SMARCD1*; tubulin folding cofactor D gene *TBCD*; mediator complex subunit 31 *Med31*; ribosomal protein S14 *Rps14* [63], *hb* [64], *MpMIF1* [59] and *Aquaporin* gene *MpAQP1*; sucrase gene *MpSUC1*; and sugar transporter gene *MpSt4* [65], resulted in significant reduction in the target gene expression and reduced fecundity. Aside from plant-mediated silencing, several cases in which plants expressing lectins for controlling *M. persicae* were reported and those include *Galanthus nivalis* agglutinin, *GNA* expressing tobacco and potato plants [66,67], *Allium sativum* leaf lectin, *ASAL* [68], *Allium cepa* agglutinin, *ACA* expressing mustard [69], *Pinellia ternate* agglutinin, PTA expressing tobacco plants [70,152], *Dioscorea batatas* tuber lectin 1, *DB1* [71], legume lectins: *ConA* expressing potato plants [72], Jacalins: *Helianthus tuberosus* agglutinin, *HTA* expressing tobacco plants [73] and *NICTABA*-related lectin, *AtPP2* expressing in *Arabidopsis* [74], all showed significant insecticidal activity against *M. persicae*.

The tobacco aphid *Myzus nicotianae* is another destructive pest distributed worldwide. *M. nicotianaei* is known to transmit potyviruses, umbraviruses and polioviruses (http://www.ictvonline.org/). Although silencing genes of this aphid species have not been reported, transgenic plants using lectins were developed against tobacco aphids and the plants showed resistance. Tobacco transgenic plants using ASAL [75], *Zephyranthes grandiflora* agglutinin, ZGA [76], *Pinellia pedatisecta* agglutinin, PPA [77] and *Sambucus nigra* agglutinin, SNA-I’ [78] showed significant insecticidal activity against this aphid species, suggesting that using silencing plants against aphid genes has the potential for pest control using this strategy.

*Aphis gossypii* is a small aphid distributed worldwide and having host range of about 90 plant families and causes leaf yellowing and weakens plants [153]. *A. gossypii* transmits plants viruses belonging to the families *Caulimoviridae* and *Luteoviridae*. In 2014, Gong et al. [79] demonstrated the knockdown of carboxylesterase gene *CarE* in *A. gossypii* by oral feeding dsRNA-*CarE* (100 ng/µL) and the results showed 33% reduction in gene expression. Suppression of *CarE* expression increased susceptibility to omethoate. This study indicated that *CarE* is a major target for organophosphate (OPs) resistance in *A. gossypii*. Similarly, Over-expression of Cytochrome P450 monooxygenase gene *CYP6DA2* increased sensitivity to spirotetramat, alpha-cypermethrin and the toxicity of gossypol to cotton aphids [80,81]. Another study has demonstrated the silencing of the odorant-binding protein 2 (*OBP2*) which resulted in impaired host-seeking and oviposition behavior of *A. gossypii* [82]. Furthermore, cotton transgenic plants expressing *Amaranthus caudatus* agglutinin (amaranthin) conferred enhanced resistance to *A. gossypii* [83].

The grain aphid *Sitobion avenae* causes serious damage in cereal crops, especially wheat, by direct feeding and indirectly by transmitting members of plant viruses belonging to the *Luteoviridae* and *Potyviridae* [53]. Approaches combining different methods in knocking down *S. avenae* genes using RNAi were demonstrated and the results were remarkable. For example, silencing cytochrome *c* oxidase subunit VIIc precursor, zinc finger protein and three unknown proteins [84] and secreted salivary peptide *DSR32*, salivary protein *DSR33*, serine protease 1 *DSR48* [85] resulted in high mortality rates. Another study has shown silencing of *S. avenae* catalase *CAT* gene which significantly influenced its survival [86]. Oral delivery of *olfactory receptor* dsRNA [87] resulted in mortality and induced wing morph differentiation in *S. avenae*, while injection of dsRNA of *Ace1* reduced fecundity [88]. More recently, Wang et al. (2015) [85] performed de novo transcriptome sequencing of grain aphid and out of 66 unigenes selected for dsRNA artificial diet assays, four of these genes: *cytochrome c oxidase subunit VII c precursor*, *secreted salivary peptide*, *salivary protein MYS2* and *serine protease 1* caused high mortality rates among treated aphids. Silencing of Carboxylesterase gene *CbE E4* [90] and Acetylcholinesterase gene *Ace1* [88] impaired their tolerance to insecticides. Similarly, Bird cherry-oat aphid *Rhopalosiphum padi* which transmits potyviruses was fed with *Ace1* dsRNA resulting in increased susceptibility to insecticides (pirimicarb and malathion) and reduced fecundity [88]. Furthermore, transgenic plants with *GNA* lectin reduced nymph production by 46.9% [89].

The green bug *Schizaphis graminum* is a major pest of small grains and causes damage by direct feeding which induces phytotoxic responses that directly influence the crop yield [53]. *S. graminum* transmits Barley yellow dwarf virus (BYDV, *Luteovirus*) and Cereal yellow dwarf virus (CYDV, *Poleovirus*), the most economically important virus diseases of cereal crops worldwide [154]. As in *A. pisum*, silencing *C002* in *S. graminum* caused significant lethality of the insect [91]. Similarly, transgenic plants expressing PTA also showed significant insecticidal activity against the insect [92].

*Lipaphis erysimi* is the mustard aphid and transmits turnip mosaic potyviruses [155]. Plants expressing dsRNA against the genes *GNA*, *ASAL*, *ACA* [69,93] and wheat germ agglutinin, *WGA* [94] showed significant insecticidal activity against this aphid species. However, among them, *ACA*-expressing mustard plants were found to be most toxic to the mustard aphids [69]. Another insect vector, *Aulacorthum solani*, known to transmit Soybean dwarf virus (SbDV) [156], showed decreased fecundity when exposed to *GNS*-transgenic plants [95].

### 4.2. Whiteflies

Whiteflies (family *Aleyrodidae*) are tiny 1 mm long sap-sucking insects that feed on hundreds of plant species and cause damage by direct feeding, and indirectly by transmitting plant viruses. A total of five whitefly spp (*Bemisia tabaci*, *Bemisia afer*, *Trialeurodes vaporariorum*, *T. abutiloneus* and *T. ricini*) are known to transmit plant viruses in the genera *Begomovirus*, *Carlavirus*, *Crinivirus*, *Ipomovirus*, and *Torradovirus* [157,158]. During the past two decades, *B. tabaci* (Gennadius) (Hemipetra: Aleyrodidae) has been considered one of the most economically important insect–vector complexes, threatening major crops worldwide. *B. tabaci* is the most important whitefly species as phloem-feeder and virus vector. This insect transmits more than 280 plant viruses that belong to the genera *Begomovirus*, *Crinivirus*, *Carlavirus*, *Torradovirus* and *Ipomovirus*, and known as one of the most successful insects that developed resistance to all major insecticide classes [157,158].

The application of RNAi technology has been demonstrated in whiteflies and showed promising results to be used as a new approach, pesticide-free method to control whiteflies. First report about the RNAi machinery being present in whiteflies was by Ghanim et al. (2007) [37]. In this study, the authors demonstrated that injection of dsRNA resulted in significant reduction in the expression of the targeted mRNAs in different organs of the insect. Introducing dsRNA and siRNAs of actin ortholog into the insect for *ADP/ATP translocase*, *alpha-tubulin*, *ribosomal protein L9* (*RPL9*), and *Vacuolar-type ATPase A subunit* caused 29%–97% mortality after six days of feeding [96]. Similarly, a study showed efficient silencing of the *P450 CYP6M1* gene which reduced gene expression, increased mortality and reduced the ability of the insect to detoxify imidacloprid and nicotine in both MEAM1 and MED biotypes [97].

*B. tabaci* feeding on transgenic tobacco plants expressing dsRNA against *v-ATPase A* [98] and the osmoregulators *aquaporin*, *AQP* and *alpha glucodiase*, *AGLU* [99], showed significant reduction in transcript levels of the target genes in the insect, and caused various levels of mortality. Recently, it was demonstrated that expressing dsRNA of whitefly in the entomapthogenic fungus *Isaria fumosorosea*, successfully silenced the insect *Toll-like receptor 7* (*TLR7*) gene by infecting nymphs and increased the mortality rate [45].

Plant-mediated gene silencing also showed effects on whitefly fitness and development. Suppressing the terpenoid synthesis in tobacco plants via gene silencing improved whitefly fitness and favored vector–virus mutualism [100]. Similarly, silencing protein kinase (*GhMPK3*) by VIGS resulted in suppression of the MPK-WRKY-jasmonic acid (JA) and ethylene (ET) pathways and resulted in enhanced whitefly susceptibility and significant effects on eggs and pupa [101].

### 4.3. Planthoppers

Planthoppers are among the most important vectors that transmit plant viruses and approximately transmit 3% of all plant viruses (*Tenuivirus*, *Nucleorhabdovirus*, *Fijivirus*, *Phytoreovirus*, and *Oryzavirus*) worldwide [1]. Planthoppers (Family *Delphacidae*) transmits viruses to the Poaceae including maize, rice, sugarcane, Sorghum and wheat.

The brown planthopper (BPH) *Nilaparvata lugens* is the best known plant virus vector in southeast Asia and transmits both rice grassy stunt (RGSV) and rice ragged stunt (RRSV) viruses in rice in a persistent manner [159]. An efficient and convenient RNAi technique was demonstrated by silencing *N. lugens* genes through various delivery methods including injections (*calreticulin*, *cathepsin-B*, *beta2*) [102], artificial feeding (*trehalose phosphate synthase*, *NITPS*) [41]/ingestion of *V-ATPase-E*, *21E01* [103]. Further studies demonstrated silencing by feeding on transgenic plants expressing dsRNA of three *N. lugens* midgut expressing genes, the *hexose transporter gene HT1*, the *carboxypeptidase gene* (*CAR*) and the *trypsin-like serine protease gene* (*TRY*). The expression of the three genes was reduced, however, in the midguts, no phenotypic effects were observed [47]. Knockdown of *N. lugens NlFoxA* significantly decreased the number of offspring and had significant impact on the development of *N. lugens* ovaries [104]. Silencing the ecdysone receptor in three planthopper virus vectors: *N. lugens*, *Laodelphgax striatellus*, vectors for Rice stripe virus (RSV) and *Sogatella furcifera*, the vector of Southern rice black-streaked dwarf virus (SRBSDV), resulted in phenotypic defects in molting nymph mortality [105]. Silencing of *coronatine insensitive1* (*COI1*) in rice plants increased their defense response and increased *N. lugens* susceptibility to feeding on these plants [106]. Silencing chitin synthases (*CHS1* and *CHS1a*) resulted in elongated distal wing pads and crimpled cuticle phenotypes and eventually led to insect lethality; whereas the phenotypes caused by injection of *CHSb* showed increased mortality [107]. Xue et al. (2013) [108] demonstrated that silencing genes associated with flight: *indirect flight muscle* (IFM) and *dorsal longitudinal muscle* (DLM) in BPH affected flight. Xu et al. (2013) [109] comprehensively investigated the repertoire of core genes involved in siRNA and miRNA pathways in the BPH and the results demonstrated that the miRNA pathway was involved in BPH metamorphosis as depletion of the *Argonaute* (*AGO1*) or *Dicer* (*Dcr-1*) genes severely impaired ecdysis. RNAi experiments using the *N. lugens Dcr-2* gene showed 55% decrease of the gene expression after four days of feeding and no developmental changes were observed in the insect [110].

RNAi knockdown of *glutamine synthase* (*GS*) gene reduced fecundity of *N. lugens* by 64.6%, disrupted ovary development and inhibited *vitellogenin* (*Vg*) expression [111]. Zhang et al. (2013) [112] demonstrated that *Dcr-1* was crucial for the regulation of oogenesis in telotrophic ovary in *N. lugens*. Similarly, silencing of *Hsp70* and *Arginine kinase* (*Argk*) are essential for survival and triazophos increased thermotolerance in BPH [113]. RNAi targeting two *N. lugens* GST genes, *GSTe1* and *GSTm2*, significantly increased the sensitivity of the fourth instar nymphs to chlorpyrifos [114]. *N. lugens* feeding on transgenic rice plants expressing *ecdysone receptor* (*EcR*) dsRNA significantly reduced the survival rate of the offspring [115]. Lu et al. (2015) [116] also suggested that using RNAi against Vg receptor is crucial for Vg uptake into oocytes that influence insect fecundity.

Silencing the enolase gene (*Eno1*) from *N. lugens* showed significant down-regulation of the mRNA levels along with decreased egg lay and population size in the next generation [117]. In the same insect, RNAi of *Bicaudal-C* suggested its role in oogenesis and oocyte maturation [118]. In another study, silencing *acyl-coenzyme A oxidase* (*ACO*) [119,120] and *glutamine synthetase* (*GS*) [121] decreased the reproduction and population growth in BPH females, while knockdown of *NIHsp90* by dsRNA injection reduced the survival and verified its role in thermotolerance [122]. Another study demonstrated the importance of β-*N-acetylhexosaminidase* gene family in BPH and the silencing of this gene caused molting failure and death [123]. Yang et al. (2016) [124] demonstrated that knockdown of two trehalose-6-phosphate synthases (*TPS1* and *TPS2*) severely affected chitin metabolism and increased molting deformities and mortality rates, and showed that the nutritional signaling regulates Vitelogenin synthesis and egg development in *N. lugens* [125,126]. Finally, using gene silencing, Bao et al. (2016) [127] demonstrated the role of P450 proteins (*CYP6AY1* and *CYP6ER1*) in imidacloprid resistance and suggested that *CYP6ER1* gene could be induced by imidacloprid.

High mortality rates in *L. striatellus*, a small planthopper that feeds and damages rice by sap-sucking and transmitting plant viruses, were obtained when *chitinase* was silenced [128], while knocking down the Halloween gene *Shade* (*ShD*) decreased the expression of *EcR* gene and caused nymphal lethality and delayed development [160]. Knockdown of the Halloween gene *disembodied* (*dib*) in this insect delayed nymphal growth and caused mortality [129], while gene silencing by dsRNA feeding showed that the cytochrome P450 monooxygenase *CYP353D1v2* could significantly enhance the sensitivity of *L. striatellus* to imidacloprid [130].

Silencing *wingless* in the white-backed planthopper *S. furcifera*, which feeds on rice and especially attacks the seedling stage and transmits several plant viruses, resulted in significantly shorter and deformed wings [133]. Knockdown of the *disembodied* (*dib*) gene in this insect delayed nymphal growth and caused high mortality rates [129]. Introduction of dsRNA of the Halloween gene *phantom* (*phd*) in the diet of second instars successfully knocked down the expression levels of ecdysteroid hormones ecdysone (E) and 20-hydroxyecdysone (20E), and caused mortality, while slowing down ecdysis during the nymphal stages [161]. In another study, dietary ingestion of ryanodine receptors (*RyR1* and *RyR2*) dsRNA significantly reduced the mRNA level of *RyR* in the treated nymphs by 77.9% and 81.8% respectively, and greatly decreased chlorantraniliprole-induced mortality [134]. Dietary introduction of dsRNA of the Halloween gene *spook* (*spo*) to the second instars successfully reduced the target gene and reduced expression level of *Ecr*, caused mortality and delayed development during nymphal stages of *S. furcifera* [135].

### 4.4. Leafhoppers

Leafhoppers are plant feeders, known to transmit plant viruses and phytoplasma in many important crops. They belong to the family *Cicadellidae* and *Delphacidae* and transmit semi-persistent, persistent, and propagative viruses. Those viruses belong to several families that include *Bunyaviridae*, *Geminiviridae*, *Reoviridae* and *Rhabdoviridae* [1].

The green rice leafhopper *Nephotettix*, is an important rice pest in Asia and an efficient vector of the virus that cause the tungro disease. Recently, RNAi has been successfully demonstrated in *Nephotettix cincticeps*. Silencing was achieved by injecting dsRNA of *laccase-2* into first instar nymphs, which resulted in high mortality rates and depigmentation of the side lines on the body [137]. Silencing was also demonstrated in the black-faced leafhopper *Graminella nigrifrons*, known to be the only vector of the persistent propagative Maize fine streak virus (MFSV). Knockdown of *peptidoglycan recognition protein* (*PGRP-LC*) in this insect resulted in significant mortality that reached more than 90% [140]. In the beet leafhopper, *Circulifer tenellus* known to transmit cutoviruses that cause diseases in sugar beet, beans and other important crops, successful silencing was demonstrated by reducing the expression of *hexamine*, resulting in significant reduction in phenoloxidase-like activity and increased mortality [142].

### 4.5. Thrips

Thrips (Thysanoptera) are minute insects and are highly polyphagous and transmit plant viruses that belong to the genera *Carmovirus*, *Ilarvirus*, *Sobemovirus* and *Tospovirus* [1]. *Frankliniella occidentalis* is the most important thrips species distributed worldwide and vectors *Tomato spotted wilt virus* (TSWV). Silencing *V-ATPase-B* gene of *F. occidentalis* decreased the abundance of V-ATPase-B protein and resulted in female mortality and reduced fertility [143]. When ingested, dsRNA-expressing bacterial strains, successfully competed with the wild-type microflora, and sustainably mediated systemic knockdown phenotypes that were horizontally transmitted [144].

### 4.6. Beetles

Beetles (Coleoptera) include several families: *Chrysomelidae*, *Coccinellidae*, and *Meloidae*. Besides being tissue feeders, beetles also transmit viruses belonging to genera *Bromovirus*, *Carmovirus*, *Comovirus*, *Machlomovirus*, *Sobemovirus*, and *Tymovirus* [1]. The Western corn rootworm (WCR) (*Diabrotica virgifera virgifera*) (family *Chrysomelidae*) is one of the most devastating corn rootworm species in North America which transmits Maize chlorotic mottle virus (MCMV) [162]. One of the recent approaches to manage WCR involves using gene silencing. dsRNA was injected against the *Lac2* and *CHS2* genes and resulted in the prevention of post-molt cuticular tanning and reduced chitin levels in the midgut [145]. Bolognesi et al. (2012) and Ramaseshadri et al. (2013) [31,146] successfully suppressed Snf7 ortholog (*Snf7*) transcripts by injection of corresponding dsRNA in WCR, resulting in growth inhibition and mortality. Rangasamy and Siegfried (2012) [147] demonstrated effective silencing in WCR after feeding adults with artificial diet supplemented with dsRNA for *V-ATPase*. This feeding caused up to 95% mortality within two weeks of exposure compared to the control. The same method showed effective silencing of two genes: *cysteine protease* (*RS5*) and *immunue gene* (*att1*) [148]. Knockdown of two WCR genes, *hunchback* (*hb*) and *brahma* (*brm*), caused significant decrease in the transcripts of both genes in adult females and complete arrest of egg hatching was obtained, suggesting that both genes have functions in WCR embryonic development [149]. More recently, Fishilevich et al. (2016) [150] demonstrated that silencing of the chromatin remodeling gene *ATPase* resulted in decreasing fecundity.

## 5. RNAi in Plant Virus–Insect Interactions

Very few studies were conducted to investigate the prevention of virus transmission by vectors. One representative example was conducted in leafhoppers, in which the knockdown of the non-structural viral protein *Pns10* gene strongly inhibited the formation of tubules which in turn prevented the intercellular spread and transmission of Rice dwarf virus (RDV) by *N. cincticeps* [138]. Similarly, Chen et al. (2015) [139] demonstrated knockdown of *Pns4* in *N. cincticeps* which resulted in increased replication of RDV in cultured cells of *N. cincticeps*. This result demonstrated the major role of this protein in viroplasm formation for viral replication and assembly of progeny virions during infection in *N. cincticeps*. In *G. nigrifrons*, the down-regulation of *PGRPs* in MFSV, suggested its possible interaction with rhabdovirus transmission [141].

In planthoppers, silencing of the viral gene *P7-1* was demonstrated by using in vitro synthesized dsRNA. This treatment resulted in the disassembly of tubule and to the prevention of virus spread in the insect [136]. Another study showed that silencing *Agronaute 2* in *L. striatellus* affected Himetobi P virus (HiPV) [131]. Another study has demonstrated that silencing the planthopper *Laodelphax striatellus cuticular protein* (*CPR1*) resulted in reduced ability to transmit Rice stripe virus (RSV) by the vector [132]. In thrips, it was shown by silencing that the nonstructural (NSs) protein of TSWV aids in the replication of baculoviruses in lepidopteran cell lines [163]. This result hints on the role of this protein during TSWV transmission by thrips.

In *B. tabaci*, several studies have demonstrated the importance of insect proteins in the transmission of begomoviruses. Such examples include the small heat shock protein (BtHSP16) that was shown to interact with Tomato yellow leaf curl Sardinia virus (TYLCSV) coat protein (CP) [164], and heat shock protein 70 (HSP70) that interacts with Tomato yellow leaf curl virus (TYLCV) CP [165]. Recently, de Paula et al. (2015) [166] reported that the virus titer was reduced in whiteflies that were fed on plants expressing siRNA of the viral replication associated protein gene of Bean golden mosaic virus (BGMV).

## 6. Conclusions and Future Perspectives

In this review, we summarized known cases in which successful gene silencing in insect vectors for plant pathogens were reported. Silencing in non-model insects was in most cases designed for causing general effects such as mortality and population size control as a way for pest control. Very few cases investigated silencing genes that have direct role in virus transmission. The various delivery methods used for introducing silencing RNA molecules into the insect were successful in inducing silencing. While artificial feeding or injection are best methods for research purposes, expression of siRNA molecules in plant hosts for insect feeding and inducing silencing will be the best approach for developing means of RNAi-based pest control. Engineering plants for silencing will be effective in controlling persistent viruses, by silencing genes that have roles in the transmission process, or controlling the vector itself which can lead to the control of not only persistent viruses, but also non-persistent and semi-persistent ones. The design of transgenic plants for inducing silencing in insect vector will be ideal by using specific genes expressed in these groups of insects, and genes that are common to more than one insect pest.

## Figures and Tables

**Table 1:** Reported cases of gene silencing in insect vector for plant pathogens.

Organism	Targeted Genes	Delivery Method	Phenotype after Silencing	Reference
**Aphids**				
*Acyrthosiphon pisum*	Salivary protein *C002*	Injection	Mortality	[35]
	*C002*, *Mp10* and *Mp42*	Injection	Fecundity	[54]
	*Calreticulin*		-	[36]
	*vATPase*	Artificial Feeding	Mortality	[38]
	*Aquaporin*, *ApAQP1*	Artificial Feeding	Elevate osmotic pressure of the hemolymph	[40]
	*hunchback* (*hb*)	Artificial Feeding	Higher mortality	[29]
	*cathepsin*-*L*	Injection/Artificial Feeding	Higher mortality and impaired molting	[55]
	Angiotensin-converting 193 *ACEs*	Injection	Higher mortality	[56]
	structural sheath protein (*shp*)	Injection	Higher mortality	[57]
	Peroxiredoxin 1 gene (*ApPrx1*)	Injection	decreased survival, increased oxidative stress	[58]
	macrophage migration inhibitory factor (*MpMIF1*)	Injection	Decreased survival and fecundity	[59]
	*Cry4Aa* derived from *B. thuringiensis* subsp *israelensis*	Artificial Feeding	Higher mortality	[60]
*Myzus persicae*	*MpC002*, *MpRack1*	Tobacco and Arabidopsis	Reduced fecundity	[46]
	Effector gene *MpC002*, *MpPIntO1* and *MpPIntO2*	Tobacco and Arabidopsis	Reduced fecundity	[61]
	serine protease (*MySP*)	Arabidopsis	Reduced fecundity	[62]
	Acetylcholinesterase 2 gene *MpAChE2*; *V-ATPase E*; 40S ribosomal protein S5-like isoform-1 *Rps5*; SWI/SNF-related matrix-associated actin-dependent regulator of chromatin subfamily D member 1-like gene *SMARCD1;* tubulin folding cofactor D gene *TBCD*; mediator complex subunit 31 *Med31*; ribosomal protein S14 *Rps14*	Tobacco	Reduced fecundity	[63]
	*hunchback* (*hb*)		Reduced fecundity	[64]
	Macrophage migration inhibitory factor *MpMIF1*	Artificial Feeding	Reduced fecundity	[59]
	*Aquaporin* gene *MpAQP1*; sucrase gene *MpSUC1* and sugar transporter gene *MpSt4*	Tobacco	Reduced fecundity	[65]
	*Galanthus nivalis* agglutinin, *GNA*	Tobacco	insecticidal activity and higher resistance	[66]
		Potato	insecticidal activity and higher resistance	[67]
	*Allium sativum* leaf lectin, *ASAL*	Tobacco	insecticidal activity	[68]
	*Allium cepa* agglutinin, *ACA*	Mustard	insecticidal activity	[69]
	*Pinellia ternate* agglutinin, PTA	Tobacco	insecticidal activity	[70]
	*Dioscorea batatas* tuber lectin 1, *DB1*	Tobacco	insecticidal activity	[71]
	*ConA*	Potato	insecticidal activity	[72]
	*Helianthus tuberosus* agglutinin, *HTA*	Tobacco	insecticidal activity	[73]
	*NICTABA*-related lectin, *AtPP2*	Arabidopsis	insecticidal activity	[74]
*Myzus nicotianae*	ASAL	Tobacco	insecticidal activity	[75]
	*Zephyranthes grandiflora* agglutinin, ZGA	Tobacco	insecticidal activity	[76]
	*Pinellia pedatisecta* agglutinin, PPA	Tobacco	insecticidal activity	[77]
	*Sambucus nigra* agglutinin, SNA-I′	Tobacco	insecticidal activity	[78]
*Aphis gossypii*	*CarE*	Artificial Feeding	insecticidal activity	[79]
	Cytochrome P450 monooxygenase gene *CYP6DA2*	Cotton	insecticidal activity	[80,81]
	odorant-binding protein 2 (*OBP2*)	Cotton	impaired host-seeking and oviposition behavior	[82]
	*Amaranthus caudatus* agglutinin (amaranthin)	Cotton	insecticidal activity	[83]
*Sitobion avenae*	cytochrome *c* oxidase subunit VIIc precursor, zinc finger protein and three unknown proteins	Wheat	High mortality	[84]
	secreted salivary peptide *DSR32*, salivary protein *DSR33*, serine protease 1 *DSR48*	Artificial Feeding	High mortality	[85]
	catalase *CAT*	Artificial Feeding	Effect on its survivability	[86]
	olfactory coreceptor gene *SaveOrco*	Artificial Feeding	lethality and induced wing morph differentiation	[87]
	*Ace1*	Injection	Reduced fecundity	[88]
	cytochrome c oxidase subunit VII c precursor, secreted salivary peptide, salivary protein *MYS2* and serine protease 1	Artificial Feeding	High mortality	[85]
	*GNA*-related lectin	Maize	reduction in nymph production	[89]
	Carboxylesterase gene *CbE E4*	Wheat	impaired tolerance to insecticides	[90]
	Acetylcholinesterase gene *Ace1*	Injection	impaired tolerance to insecticides	[88]
*Rhopalosiphum padi*	Acetylcholinesterase gene *Ace1*	Injection	susceptibility to insecticides	[88]
*Schizaphis graminum*	*C002*	Artificial Feeding	Lethality	[91]
	*Pinellia ternate* agglutinin, *PTA*	Wheat	Insecticidal activity	[92]
*Lipaphis erysimi*	*Galanthus nivalis* L. agglutinin, *GNA*, *Allium sativum* L. leaf agglutinin, ASAL, *Allium cepa* L. agglutinin, *ACA*	Mustard	*insecticidal activity*	[69,93]
	*wheat germ agglutinin*, *WGA*	Mustard	*insecticidal activity*	[94]
	*Allium cepa* L. agglutinin, *ACA*	Mustard	*insecticidal activity*	[69]
*Aulacorthum solani*	*Galanthus nivalis* agglutinin, *GNA*	Potato	*decreased fecundity*	[95]
**Whiteflies**				
*Bemisia tabaci*	*Chickadee*	Injection	Mortality	[37]
	ADP/ATP translocase, alpha-tubulin, ribosomal protein L9 (*RPL9*), and Vacuolar-type ATP*ase* A subunit	Artificial Feeding	Mortality	[96]
	*P450 CYP6M1*	Artificial Feeding	Increased mortality	[97]
	v-ATP*ase* A	Tobacco	Mortality	[98]
	aquaporin, *AQP* and alpha glucodiase (*AGLU*)	Tobacco	Mortality	[99]
	Toll-like receptor 7 (*TLR7*)	*Isaria fumosorosea*	Increased mortality	[45]
	Suppressing the terpenoid synthesis	tobacco	improved whitefly fitness and favored vector–virus mutualism	[100]
	protein kinase (*GhMPK3*)	cotton	effects knockdown on eggs and pupa	[101]
**Planthoppers**				
*Nilaparvata lugens*	*calreticulin*, *cathepsin-B*, *beta2*	Injection	-	[102]
	trehalose phosphate synthase, *NITPS*	Artificial Feeding	lethality	[41]
	*V-ATPase-E*, *21E01*	ingestion	-	[103]
	hexose transporter gene *HT1*, the carboxypeptidase gene (*CAR*) and the trypsin-like serine protease gene (*TRY*)	Rice	lethal phenotypic effects	[47]
	*NlFoxA*	Artificial Feeding	effect on fecundity and development of ovaries	[104]
	ecdysone	Artificial Feeding	phenotypic defects in molting and nymph lethality	[105]
	coronatine insensitive1 (*COI1*)	rice	induced defenses	[106]
	chitin synthases (*CHS1* and *CHS1a*)	Injection	insect lethality	[107]
	*CHSb*	Injection	increased mortality	[107]
	*flightin*	Artificial Feeding	affected flight	[108]
	Dicer (*dcr*), Argonaute (*ago*),	Injection	severely impaired ecdysis	[109]
	*Dcr-2*	Artificial Feeding	no developmental changes	[110]
	glutamine synthase (*GS*) gene	Injection	reduced fecundity	[111]
	*dicer1*	Injection	regulation of oogenesis in telotrophic ovary	[112]
	*Hsp70* and Arginine kinase (*Argk*)	Artificial Feeding	survival	[113]
	*GSTe1* and *GSTm2*	Injection	sensitivity of the fourth instar nymphs to chlorpyrifos	[114]
	ecdysone receptor (EcR)	Artificial Feeding	reduced the survival rate of the offspring	[115].
	Vg receptor	Injection	fecundity	[116]
	enolase gene (*Eno1*)	Injection	decreased egg lay	[117]
	*Bicaudal-C*	Injection	role in oogenesis and oocyte maturation	[118]
	acyl-coenzyme A oxidase (*ACO*)	Artificial Feeding	decreased the reproduction and population growth	[119,120]
	glutamine synthetase (GS)	Artificial Feeding/Injection	decreased the reproduction and population growth	[121]
	*NIHsp90*	Injection	reduced survival, role in thermotolerance	[122]
	β-*N*-acetylhexosaminidase	Injection	failure of the nymphs to molt which eventually led to death	[123]
	trehalose-6-phosphate synthases (*TPS1* and *TPS2*)	Injection	chitin metabolism and increased molting deformities and mortality rates	[124]
		Injection	regulates Vitellogenin synthesis and egg development	[125,126]
	P450 proteins (*CYP6AY1* and *CYP6ER1*)	Injection	imidacloprid resistance	[127]
*L. striatellus*	chitinase gene	Artificial Feeding	High mortality	[128]
	Halloween gene Shade (*ShD*)	Artificial Feeding	delayed nymphal growth and caused lethality	[129]
	cytochrome P450 monooxygenase *CYP353D1v2*	Artificial Feeding	sensitivity of *L. striatellus* to imidacloprid	[130]
	Agronaute 2	Injection	enhanced HiPV accumulation	[131]
	cuticular protein (*CPR1*)	Injection	reducing the ability to transmit *Rice stripe virus* (RSV)	[132]
*S. furcifera*	wingless gene	Artificial Feeding	shorter and deformed wings	[133]
	*disembodied* (*dib*)	Artificial Feeding	nymphal growth and caused high mortality	[129]
	Halloween gene phantom (*phd*)	Artificial Feeding	lethality and slower ecdysis during nymphal stages	[129]
	ryanodine receptors (*RyR1* and *RyR2*)	Artificial Feeding	decreased chlorantraniliprole-induced mortality	[134]
	Halloween gene spook (*spo*)	Artificial Feeding	mortality	[135]
	*P7-1*	Injection	southern rice black-streaked dwarf virus (SRBSDV) spread inside the insect	[136]
**Leafhoppers**				
*Nephotettix cincticeps*	laccase-2	Injection	high mortality	[137]
	non-structural protein *Pns10*	Injection	transmission of the *Rice dwarf virus* (RDV)	[138]
	*Pns4*	Injection	RDV replication in cultured cells	[139]
*Graminella nigrifrons*	peptidoglycan recognition protein (PGRP-LC)	Injection	high mortality	[140]
	PGRPs	Injection	possible interaction with *Rhabdovirus* transmission	[141]
*Circulifer tenellus*	hexamine	Injection	high mortality	[142]
**Thrips**				
*Frankliniella occidentalis*	*V-ATPase-B*	Injection	mortality and reduced fertility	[143]
		bacteria	systemic knockdown phenotypes	[144]
**Beetles**				
*Diabrotica virgifera*	*Lac2 and CHS2*	Injection	*prevention of post-molt cuticular tanning and reduced chitin levels in midguts*	[145]
	*Snf7 ortholog* (*Snf7*)	Artificial Feeding	*growth inhibition and eventual mortality*	[31,146]
	*V-ATPase*	Artificial Feeding	*High mortality*	[147]
	*cysteine protease* (*RS5*) and *immune gene* (*att1*)	Artificial Feeding	*Effect on survival*	[148]
	hunchback (*hb*) and brahma (*brm*)	Artificial Feeding	embryonic development	[149]
	chromatin remodeling ATP*ase*	Artificial Feeding	reduction of fecundity	[150]
